# Supplementary material for: OTUB1 triggers lung cancer development by inhibiting RAS monoubiquitination
Source: EMBO Mol Med. 2016 Feb 8;8(3):288–303. doi: 10.15252/emmm.201505972 (PMC4772950; doi:10.15252/emmm.201505972)
Supplement: Supplementary file 1 — Appendix [file EMMM-8-288-s001.pdf]

Table of contents

Appendix Figure S1: Representative examples of RAS cellular localization.

Appendix Figure S2: OTUB1 overexpression does not affect AKT signaling and stimulates KRAS-dependent ERK1/2 activation.

Appendix Figure S3: OTUB1 expression in H1993 and A549 lung cancer cells.

Appendix Figure S1

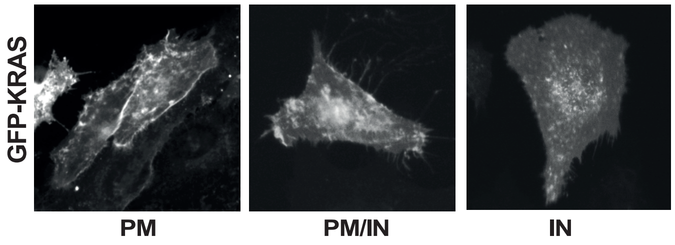

Appendix Figure S1. Representative examples of RAS cellular localization. HeLa cells expressing GFP-KRAS constructs were randomly imaged for GFP using the IN Cell Analyzer screening device. For each cell, RAS localization was scored as to be intracellular (IN), the plasma membrane (PM), or both intracellular and membrane (PM/IN).

Appendix Figure S2

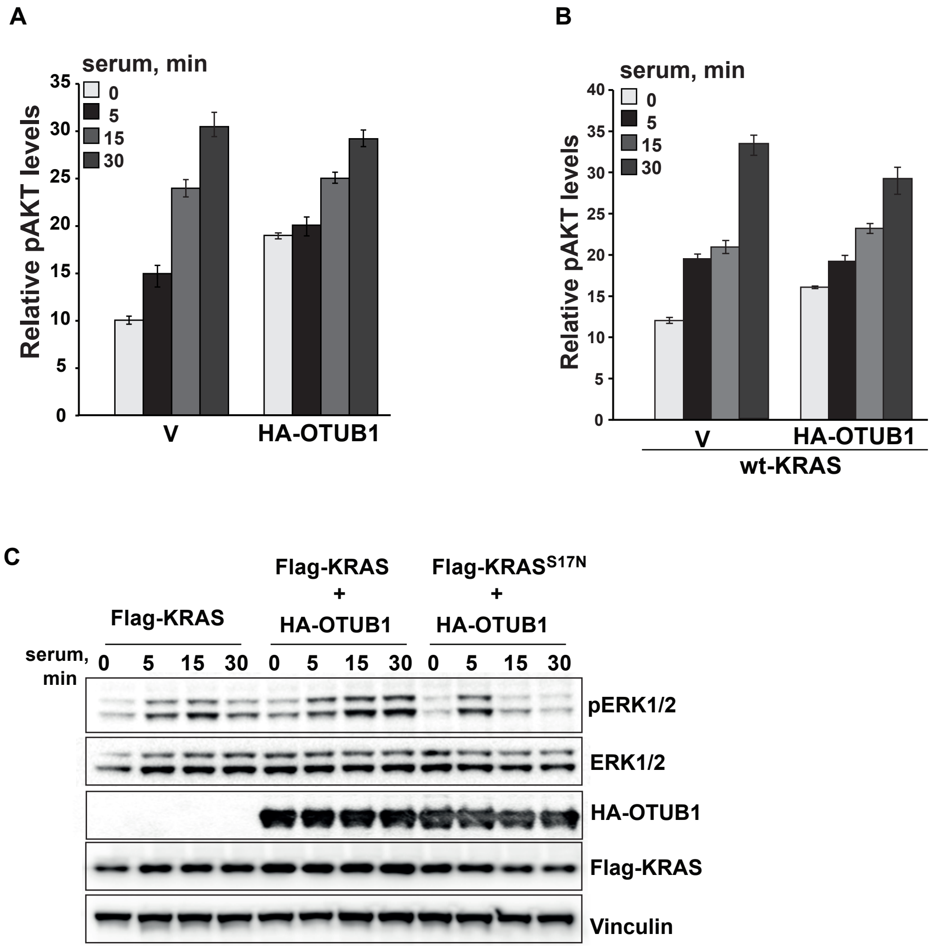

Appendix Figure S2. OTUB1 overexpression does not affect AKT signaling and stimulates KRAS-dependent ERK1/2 activation. A, OTUB1 overexpression does not affect serum-induced AKT activation. Serum-starved HEK293T cells expressing HA-OTUB1 or empty vector (V) were stimulated with 10% serum for different time periods. B, OTUB1 does not affect serum-induced KRAS-dependent AKT activation. Serum-starved HEK293T cells expressing Flag-KRAS and HA-OTUB1 or empty vector (V) were stimulated with 10% serum for the indicated time periods. **A,B,** Levels of phosphorylated AKT and total AKT were analyzed by Meso Scale assay. The results are expressed as a mean of pAKT levels relative to total AKT ± s.e.m. **C,** OTUB1 stimulates the MAPK pathway in a RAS dependent manner. Serum-starved HEK293T cells expressing wild-type KRAS or dominant-negative KRAS S17N mutant and HA-OTUB1 or empty vector (V) were stimulated with 10% serum for the indicated time periods. Whole cell lysates were analyzed by immunoblotting using the indicated antibodies.

Appendix Figure S3

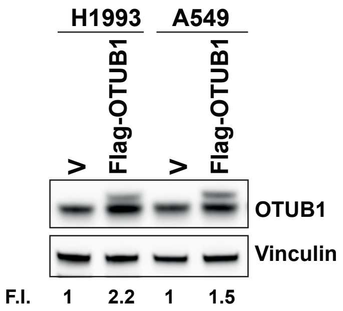

Appendix Figure S3. OTUB1 expression in H1993 and A549 lung cancer cells. Immunoblot analysis of OTUB1 expression in H1993 and A549 lung cancer cells upon lentiviral transduction of Flag-OTUB1 or empty vector (V). Up-regulation (F.I.) of OTUB1 expression relative to the control sample (V) for each cell line was quantified using ImageJ.
