# Supplementary material for: OTUB1 triggers lung cancer development by inhibiting RAS monoubiquitination
Source: EMBO Mol Med. 2016 Feb 8;8(3):288–303. doi: 10.15252/emmm.201505972 (PMC4772950; doi:10.15252/emmm.201505972)
Supplement: Supplementary file 2 — Expanded View Figures PDF [file EMMM-8-288-s002.pdf]

Expanded View Figures

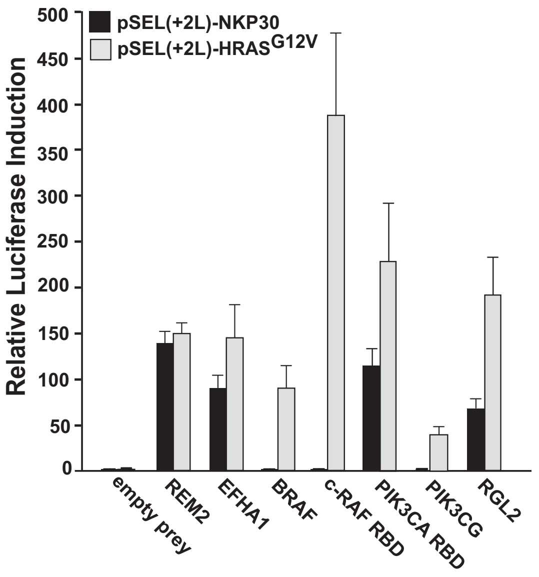

Figure EV1. MAPPIT assay to identify interacting partners of the RAS GTPases.

MAPPIT assay detected interactions between HRAS G12V and its downstream targets. A MAPPIT assay was performed with REM2 and EFHA1 as negative controls, and RAS effectors, BRAF, c-RAF RBD, PIK3CA RBD, PIK3CG, and RGL2, as preys, screened against HRAS G12V as bait. pSEL(+2L) vectors coding HRAS G12V or NKP30 negative control was expressed into HEK293T cells together with the indicated prey. The results are expressed as a mean of normalized luciferase activity ± s.e.m (leptin-treated cells vs leptin-untreated cells). $n = 2$.

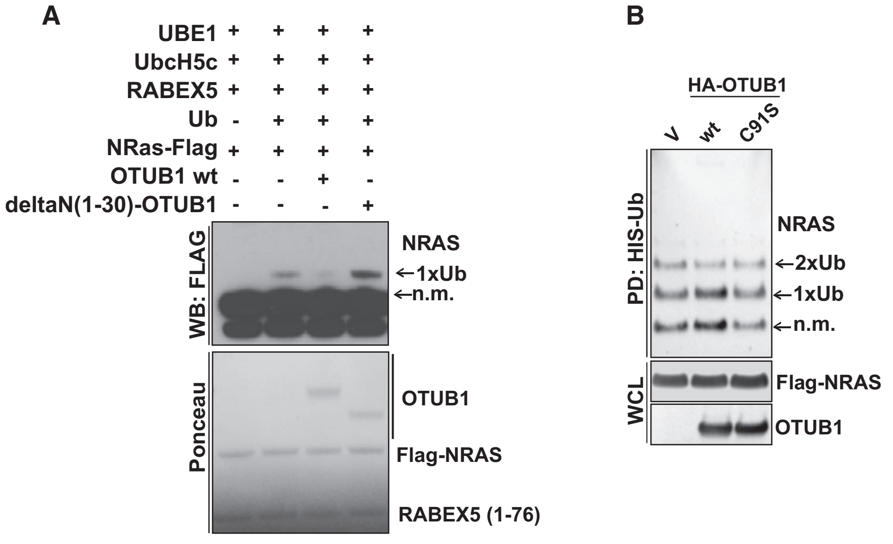

Figure EV2. OTUB1 inhibits RAS ubiquitination by suppressing E2 ligase activity.

A OTUB1 blocks RAS ubiquitination by inhibiting E2 ligase activity. Recombinant Flag-tagged NRAS was incubated with UBE1, UbcH5C (UBE2D3), ubiquitin, RABEX5 (1-76), and either wild-type OTUB1 or deltaN(1-30)-OTUB1 mutant. Ubiquitination of recombinant NRAS was analyzed by immunoblotting using anti-Flag antibody.

B OTUB1 does not de-ubiquitinate NRAS *in vitro*. The whole cell lysate overexpressing of Flag-NRAS was incubated with HA-OTUB1 wt or C91S. Ubiquitinated RAS was purified by Co^{2+} metal affinity chromatography and detected by anti-Flag antibody.

Source data are available online for this figure.

Figure EV3. OTUB1 expression is up-regulated in lung tumors.

A Copy number alterations of the OTUB1 containing region in TCGA lung adenocarcinoma and squamous cell carcinoma.

B TCGA Lung adenocarcinoma and squamous cell carcinoma were stratified according their KRAS status and OTUB1 expression levels (neg/low, medium, and high) as described in Materials and Methods.

C, D OTUB1 expression is commonly increased in lung adenocarcinomas and squamous cell carcinoma compared to matched normal lung tissues. The fold change and percent fold change of OTUB1 expression in patient tumors and matched normal tissues were determined using normalized read counts (log2-transformed).

▶

© 2016 The Authors

A

TCGA Lung Adenocarcinoma

Alteration	Patients (n=516)
Amplification (Log2 Ratio >0.4)	8 (1%)
Gain (0.1< Log2 Ratio <0.4)	128 (25%)
Mutation	14 (2%)

TCGA Lung Squamous Cell Carcinoma

Alteration	Patients (n=501)
Amplification (Log2 Ratio >0.4)	17 (3%)
Gain (0.1< Log2 Ratio <0.4)	127 (25%)
Mutation	0 (0%)

B

TCGA Lung Adenocarcinoma

	OTUB1 mRNA expression		
KRAS status	neg/low	medium	high
wt	201 (46%)	221 (50%)	19 (4%)
mutant	41 (56%)	32 (44%)	0 (0%)
Total	242 (47%)	253 (49%)	19 (4%)

TCGA Lung Squamous Cell Carcinoma

	OTUB1 mRNA expression		
KRAS status	neg/low	medium	high
wt	92 (18%)	169 (34%)	238 (48%)
mutant	1 (50%)	0 (0%)	1 (50%)
Total	93 (19%)	169 (34%)	239 (48%)

C

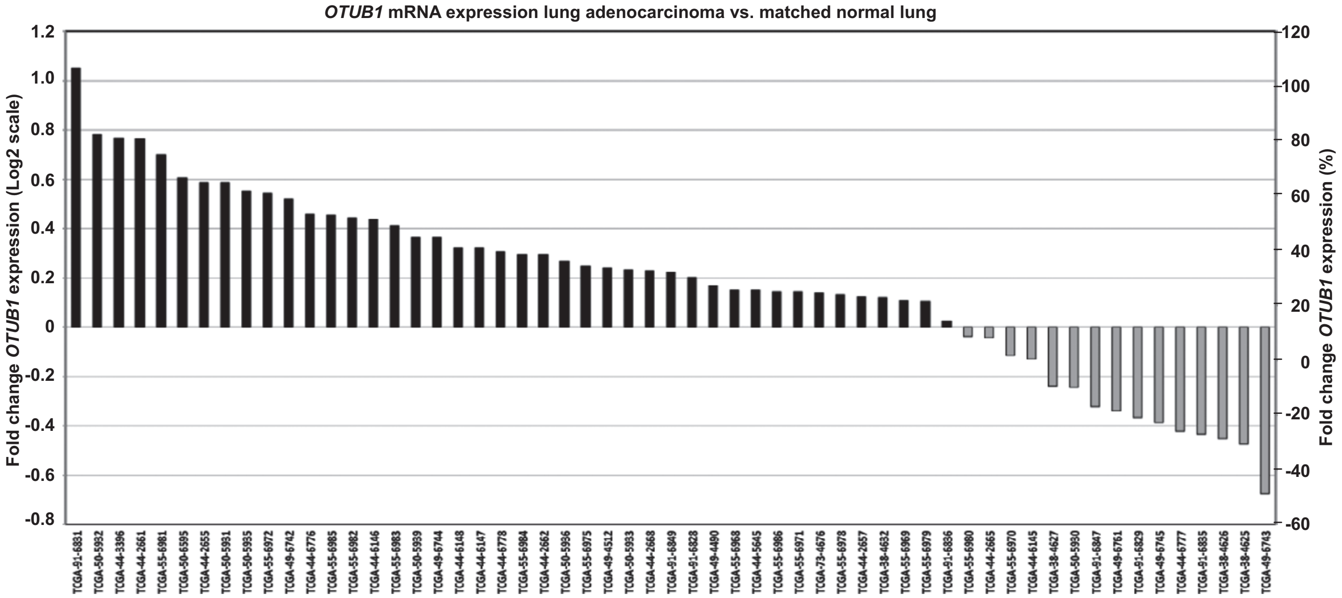

D

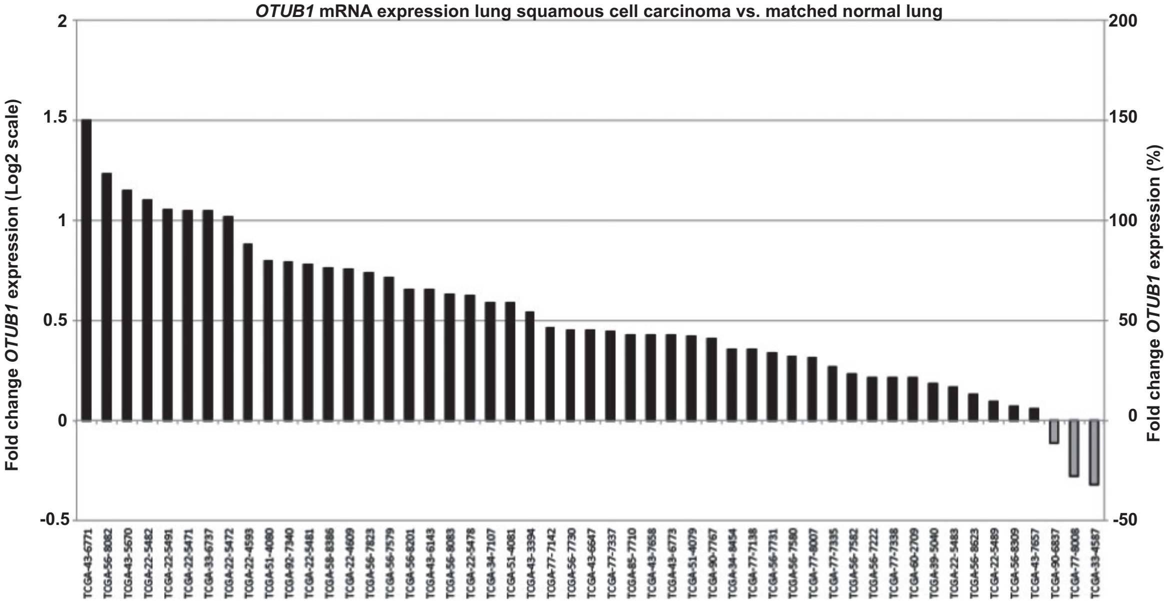

Figure EV3.

 © 2016 The Authors

A

Lung Adenocarcinoma

	OTUB1 level		
KRAS status		medium	high
wt	13 (26%)	26 (52%)	11 (22%)
mutant	2 (11%)	12 (63%)	5 (26%)
Total	15 (22%)	38 (55%)	16 (23%)

Lung Squamous Cell Carcinoma

	OTUB1 level		
KRAS status	neg/low	medium	high
wt	80 (74%)	19 (18%)	9 (8%)
mutant	0 (0%)	0 (0%)	1 (100%)
Total	80 (73%)	19 (17%)	10 (9%)

B

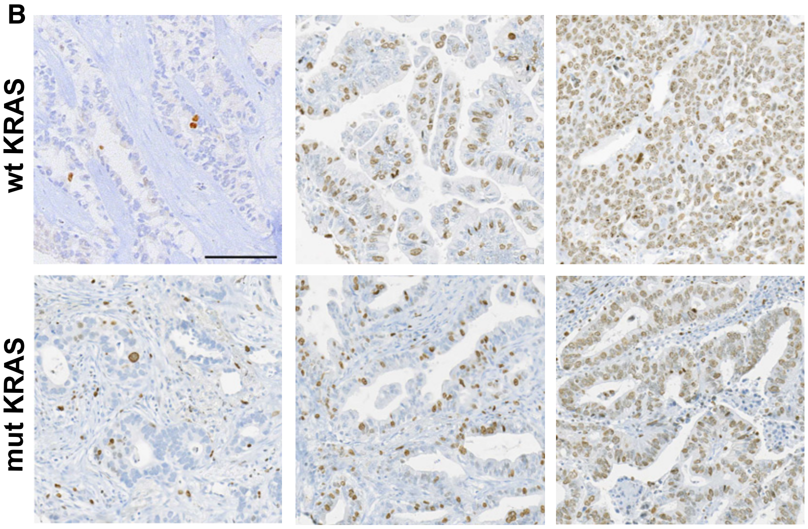

Figure EV4. IHC analysis of lung carcinoma TMAs.

A Lung adenocarcinoma and squamous cell carcinoma were stratified according to their KRAS status and OTUB1 levels (neg/low, medium, and high) as described in Materials and Methods.

B Immunohistochemical staining of Ki67 in lung adenocarcinoma. Representative images of TMA lung adenocarcinoma samples harboring wild-type or mutant KRAS immunostained for Ki67. For Ki67 scoring, the percentage of positive cells was counted and the samples scored as negative/low, less than 10% of Ki67-positive nuclei; medium, between 10 and 40% of Ki67-positive nuclei; and high, more than 40% of Ki67-positive nuclei. Scale bar, 100 μm.

© 2016 The Authors
