# Supplementary figures and images for: OTUB1 triggers lung cancer development by inhibiting RAS monoubiquitination
Source: EMBO Mol Med. 2016 Feb 8;8(3):288–303. doi: 10.15252/emmm.201505972 (PMC4772950; doi:10.15252/emmm.201505972)

Full unedited gels for Appendix Figure S2C

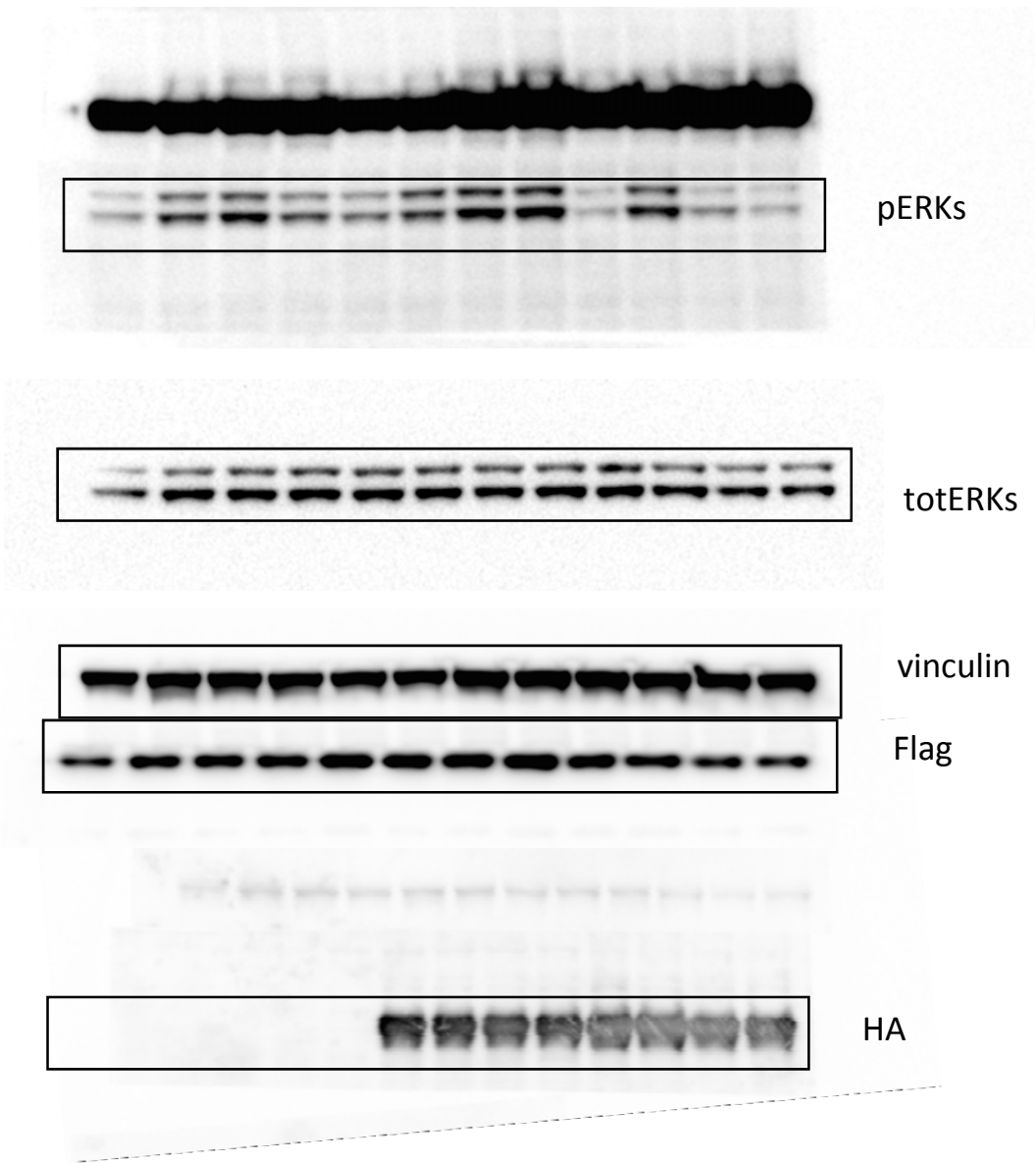

Supplement: Supplementary file 3 — Source Data for Expanded View and Appendix [file EMMM-8-288-s003.zip › Source_Data_for_Expanded_View_and_Appendix_figures/Sourc_data_for_Appendix_Figures_S2.pdf]

Full unedited gels for Appendix Figure S3

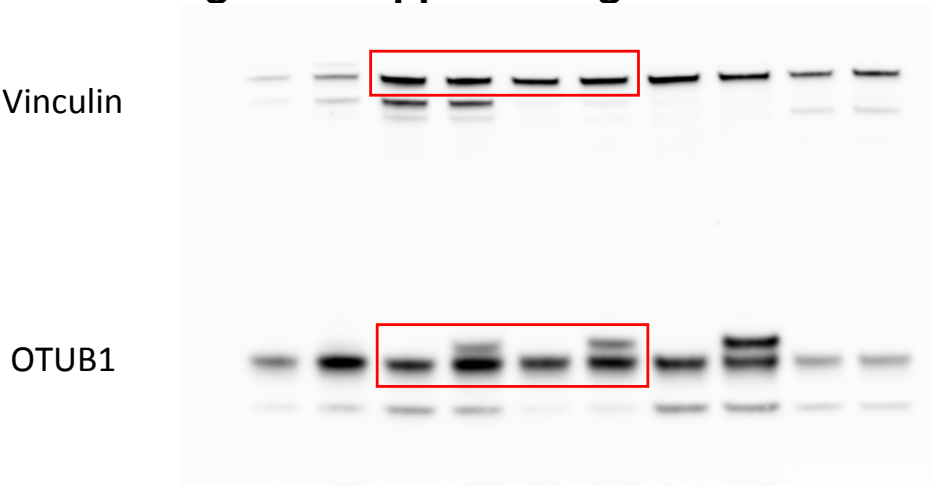

Supplement: Supplementary file 3 — Source Data for Expanded View and Appendix [file EMMM-8-288-s003.zip › Source_Data_for_Expanded_View_and_Appendix_figures/Source_data_for_Appendix_Figure_S3.pdf]

Full unedited gels for EV2A

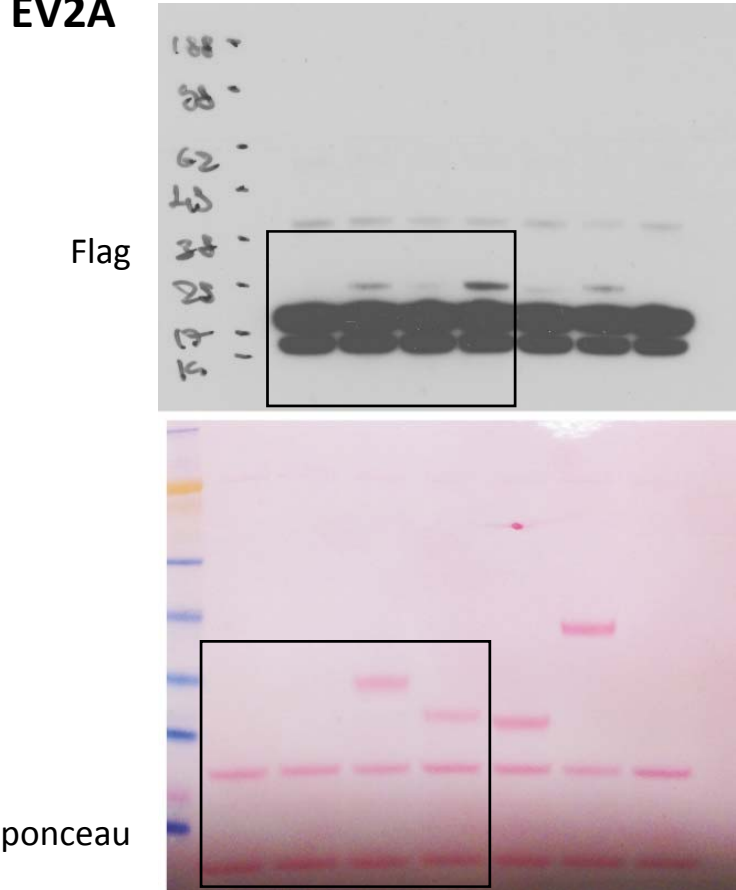

Full unedited gels for EV2B

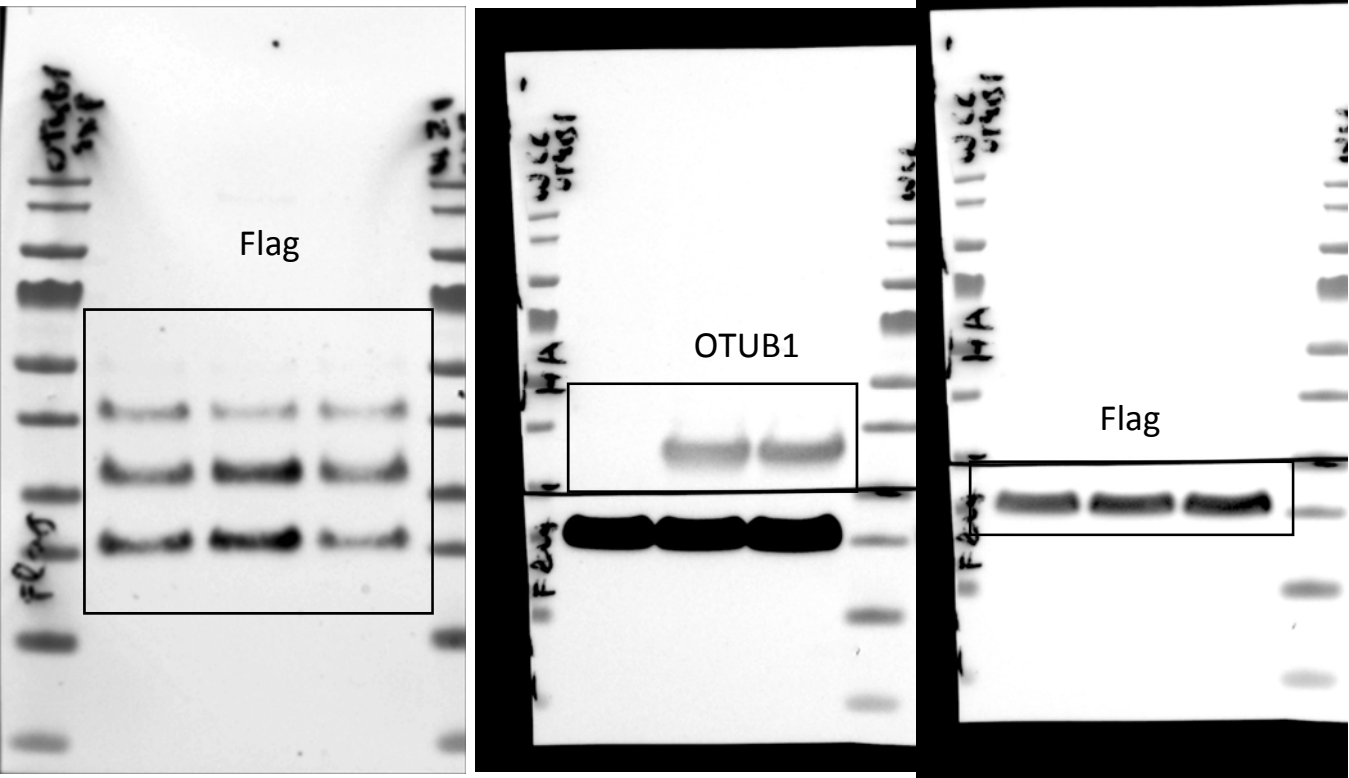

Supplement: Supplementary file 3 — Source Data for Expanded View and Appendix [file EMMM-8-288-s003.zip › Source_Data_for_Expanded_View_and_Appendix_figures/Source_data_for_EV_Figure_2.pdf]

# Full unedited gels for Figure 2A,B,C

Flag

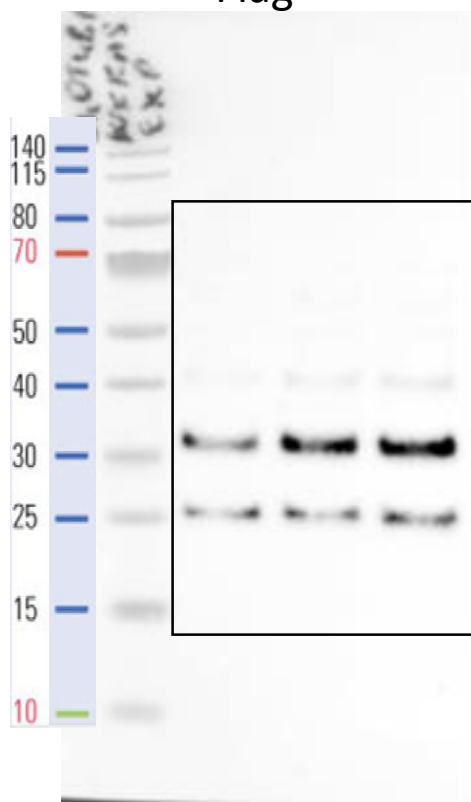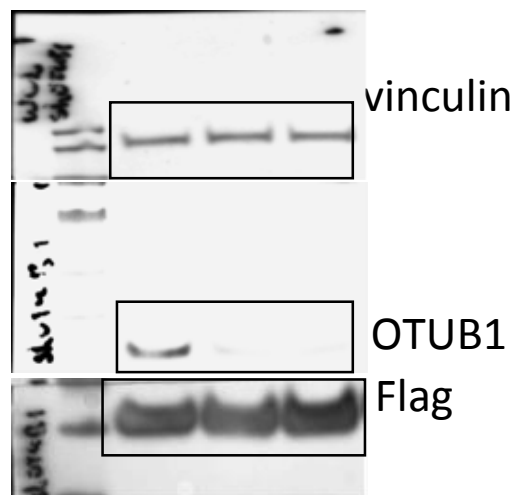

Flag

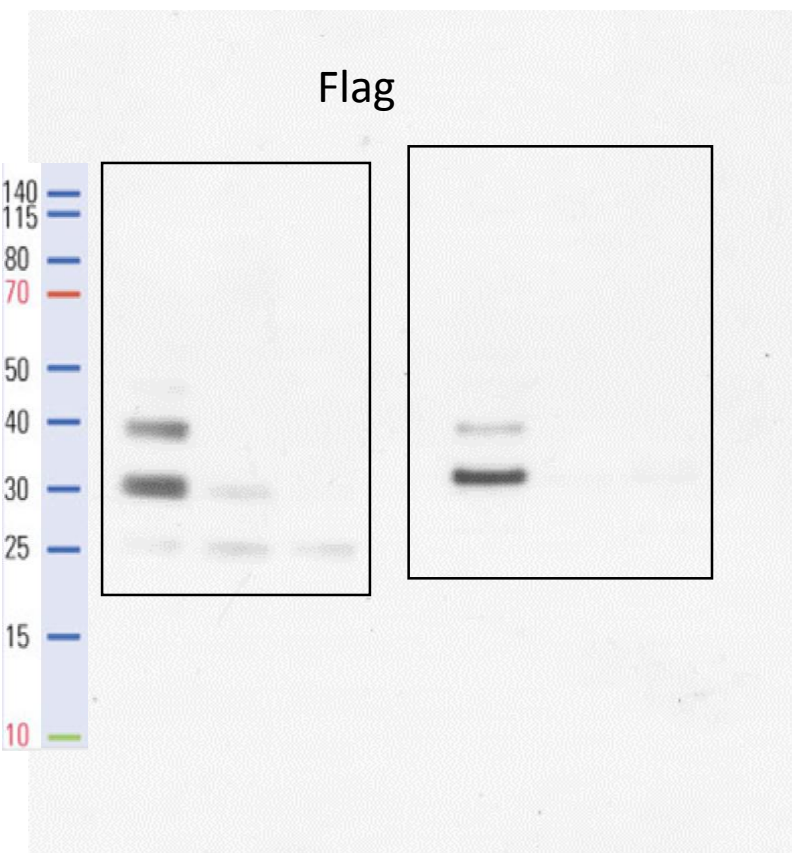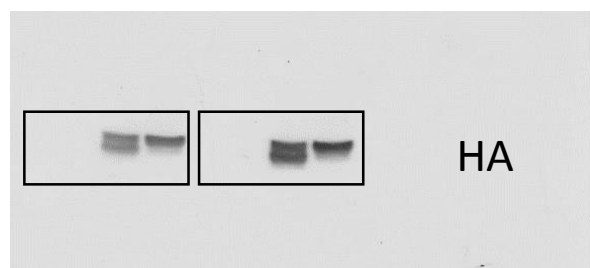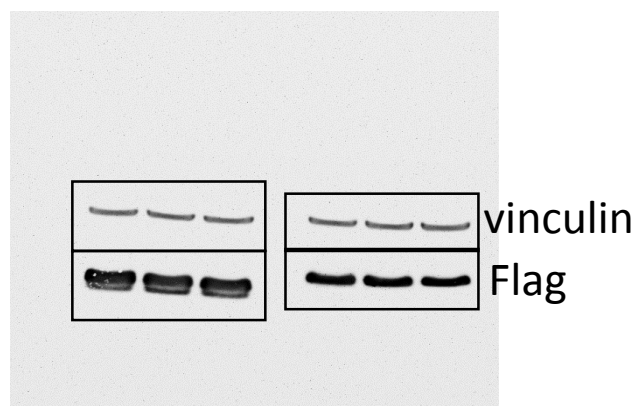

# Full unedited gel for Figure 2D

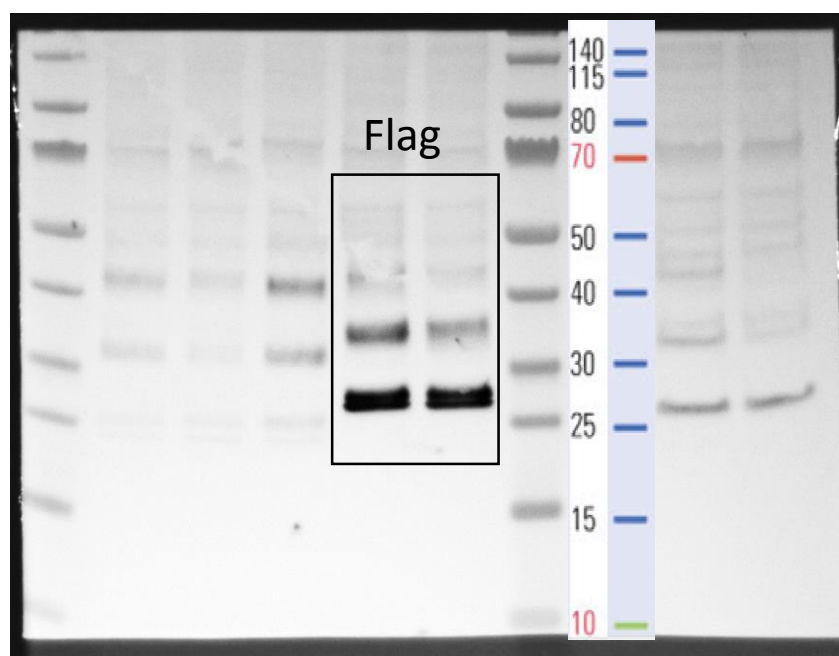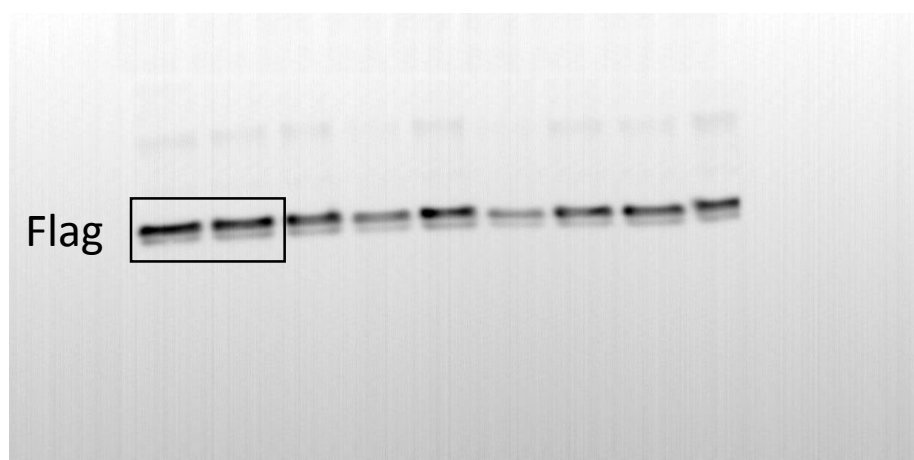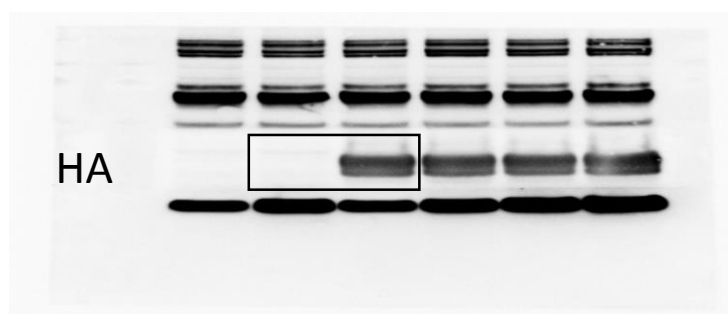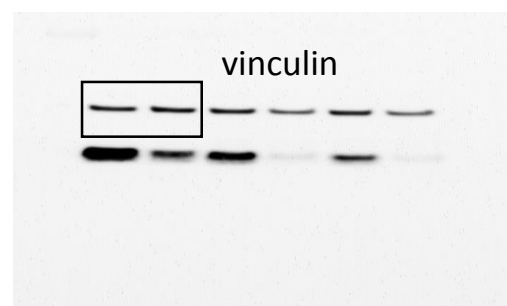

Supplement: Supplementary file 6 — Source Data for Figure 2 [file EMMM-8-288-s005.pdf]

Full unedited gels for Figure 5A

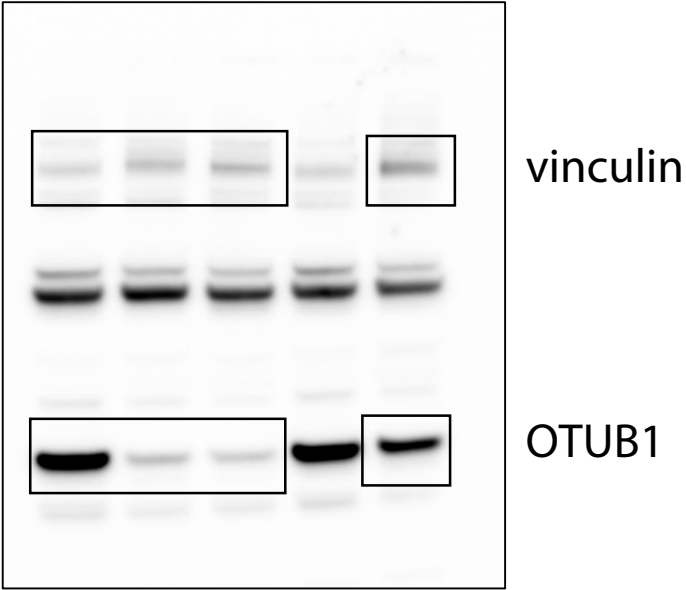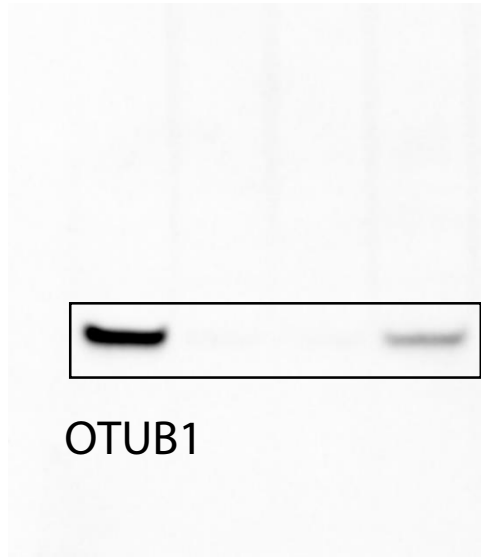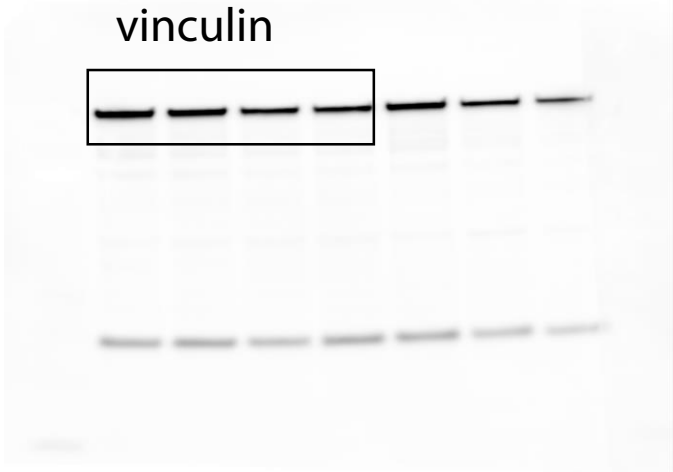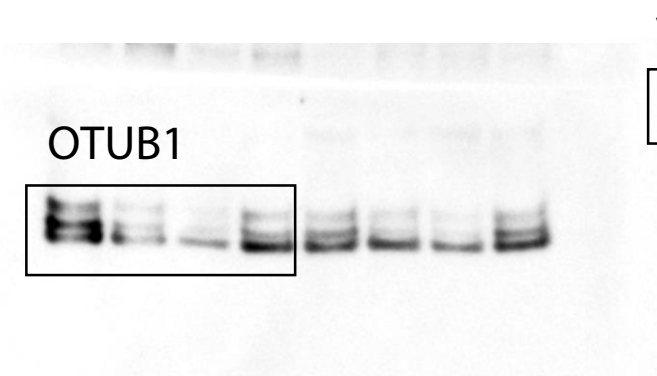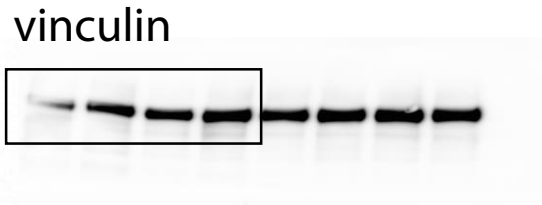

Supplement: Supplementary file 8 — Source Data for Figure 5 [file EMMM-8-288-s007.pdf]

Full unedited gels for Figure 6A

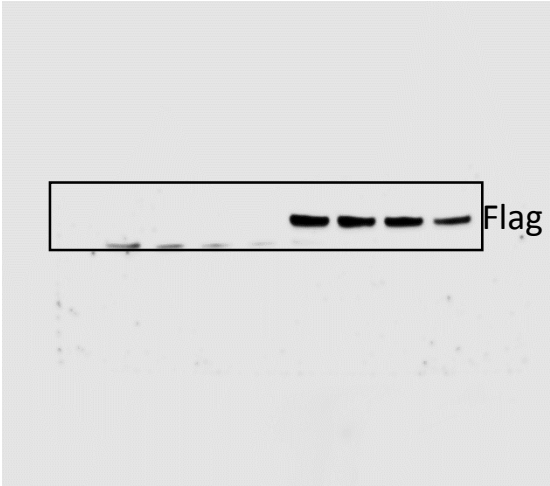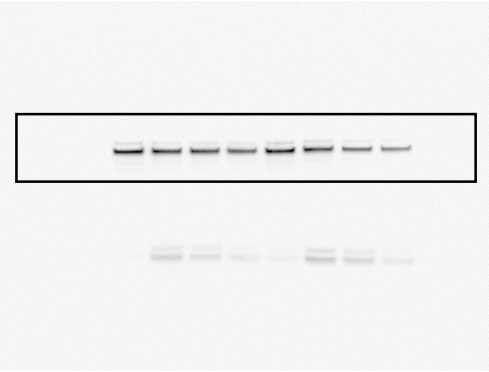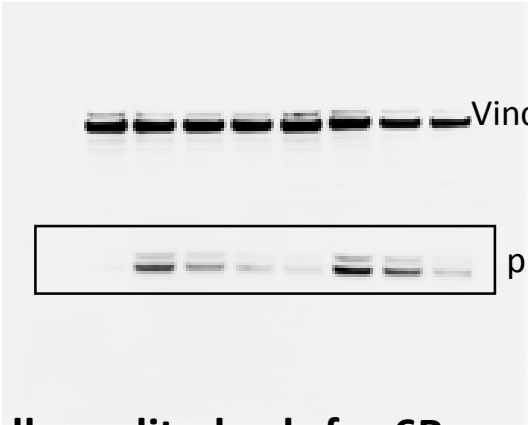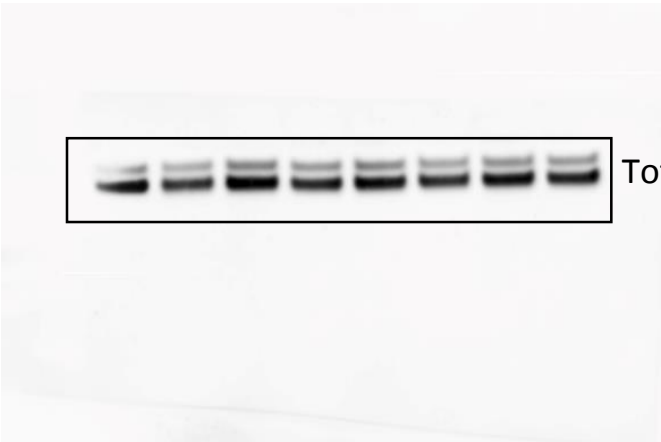

Full unedited gels for 6B

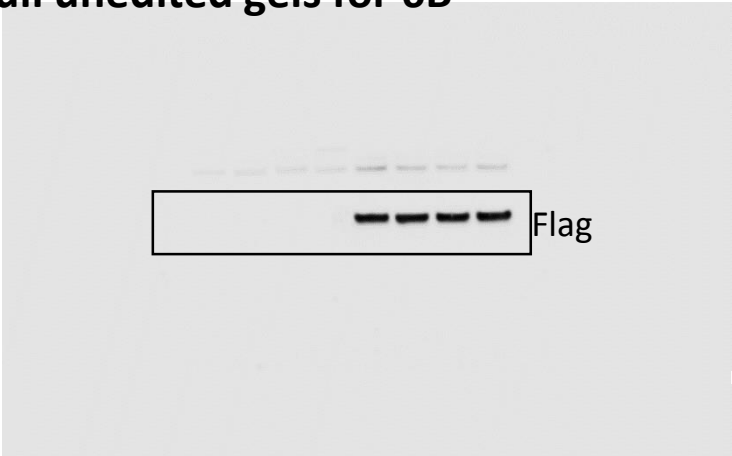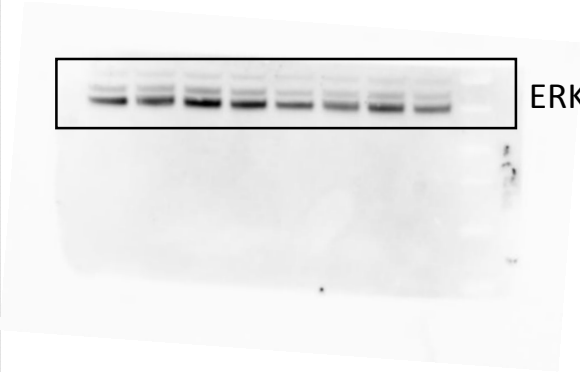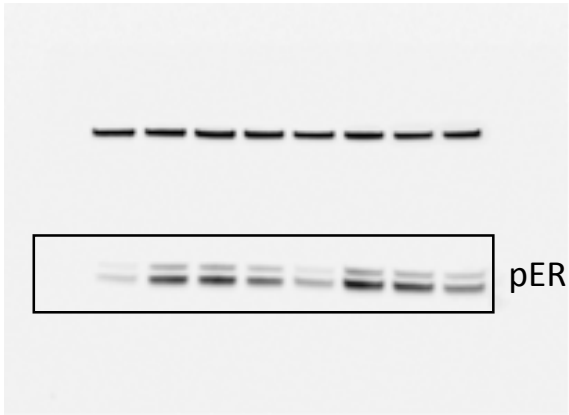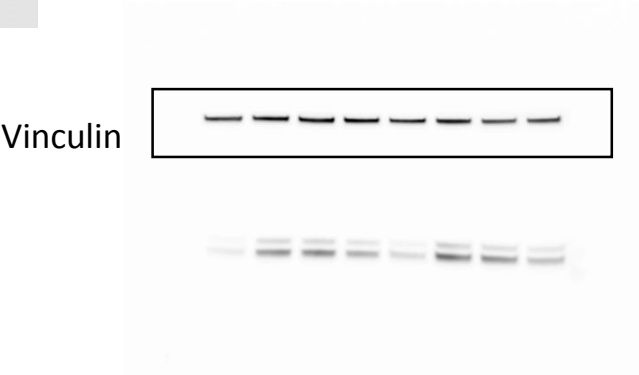

# Full unedited gels for 6C

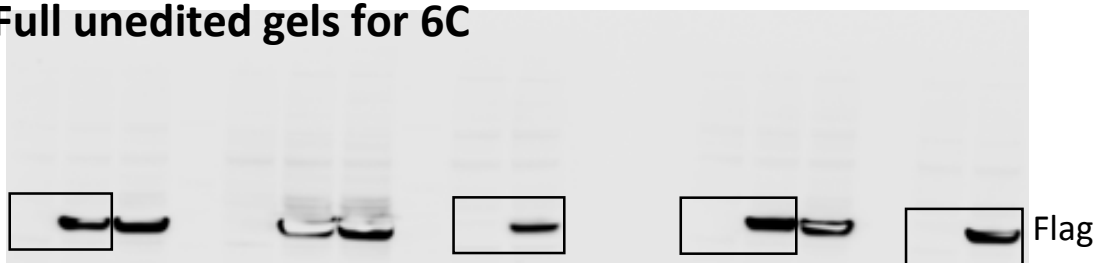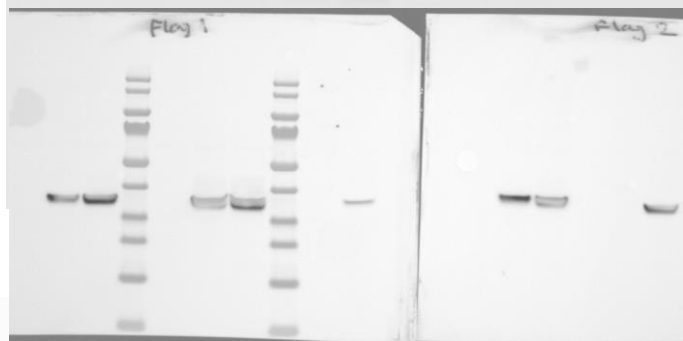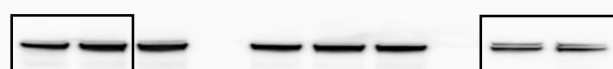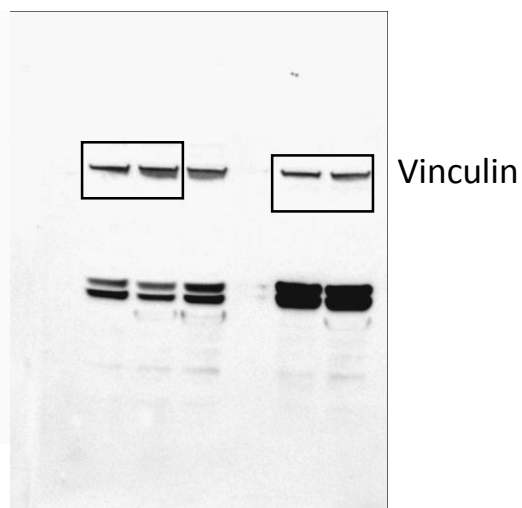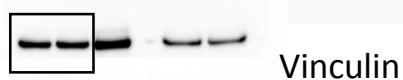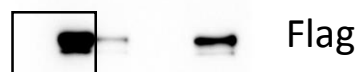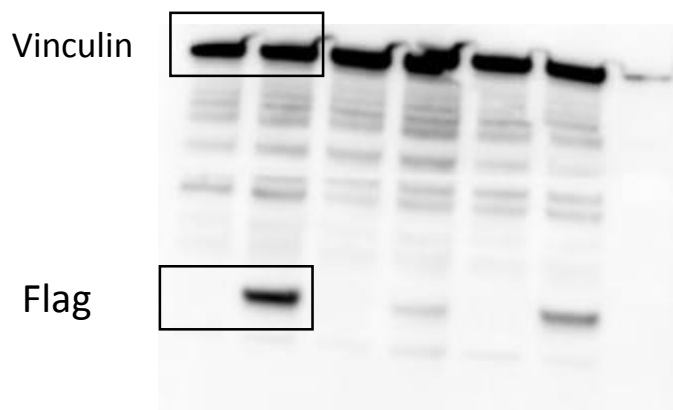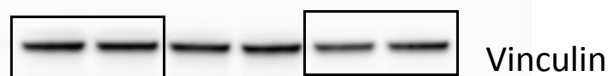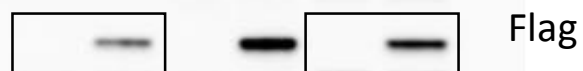

Supplement: Supplementary file 9 — Source Data for Figure 6 [file EMMM-8-288-s008.pdf]
