# Supplementary material for: OTUB1 triggers lung cancer development by inhibiting RAS monoubiquitination
Source: EMBO Mol Med. 2016 Feb 8;8(3):288–303. doi: 10.15252/emmm.201505972 (PMC4772950; doi:10.15252/emmm.201505972)

Full unedited gels for Figure 1C,D,E

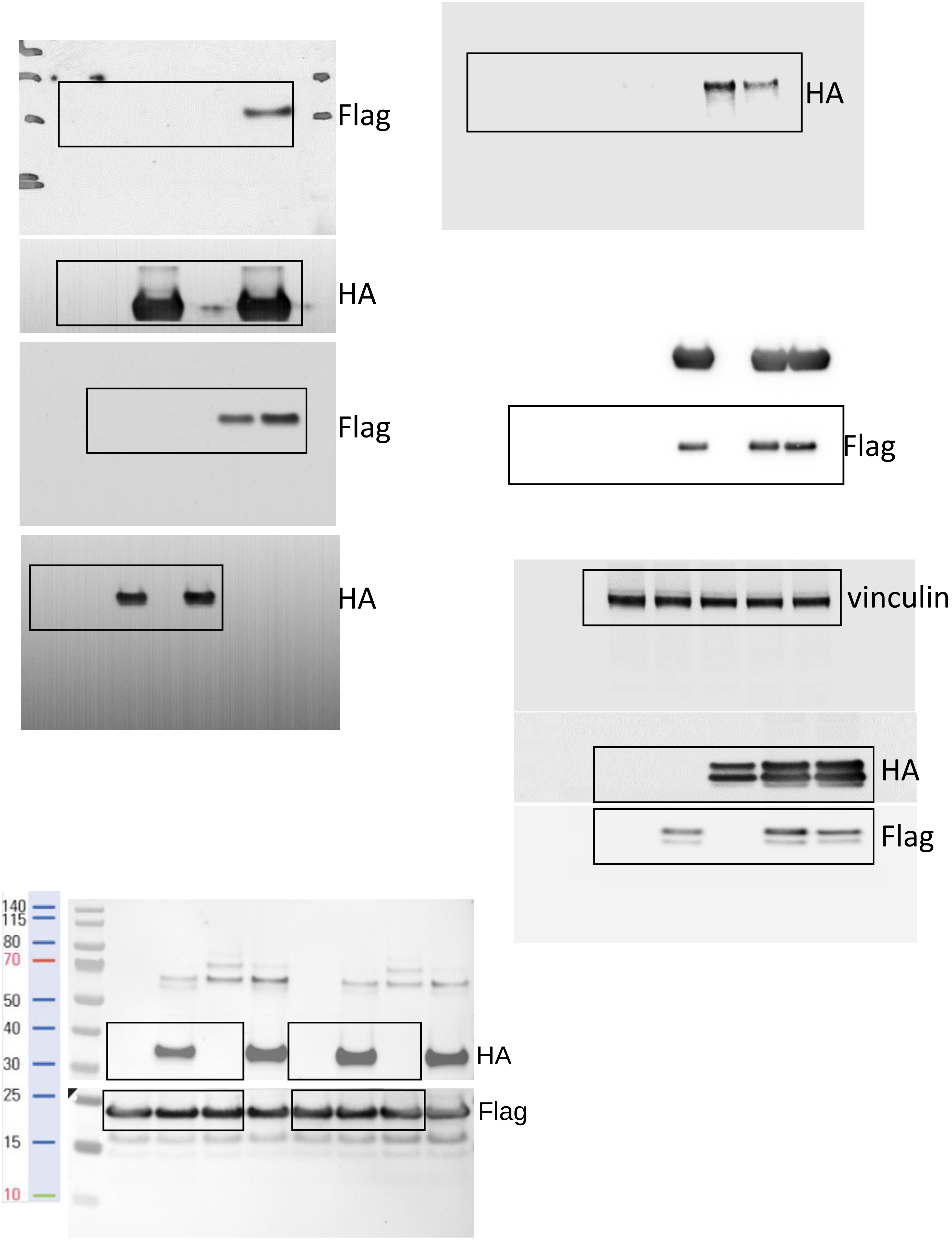

Full unedited gels for Figure 1F

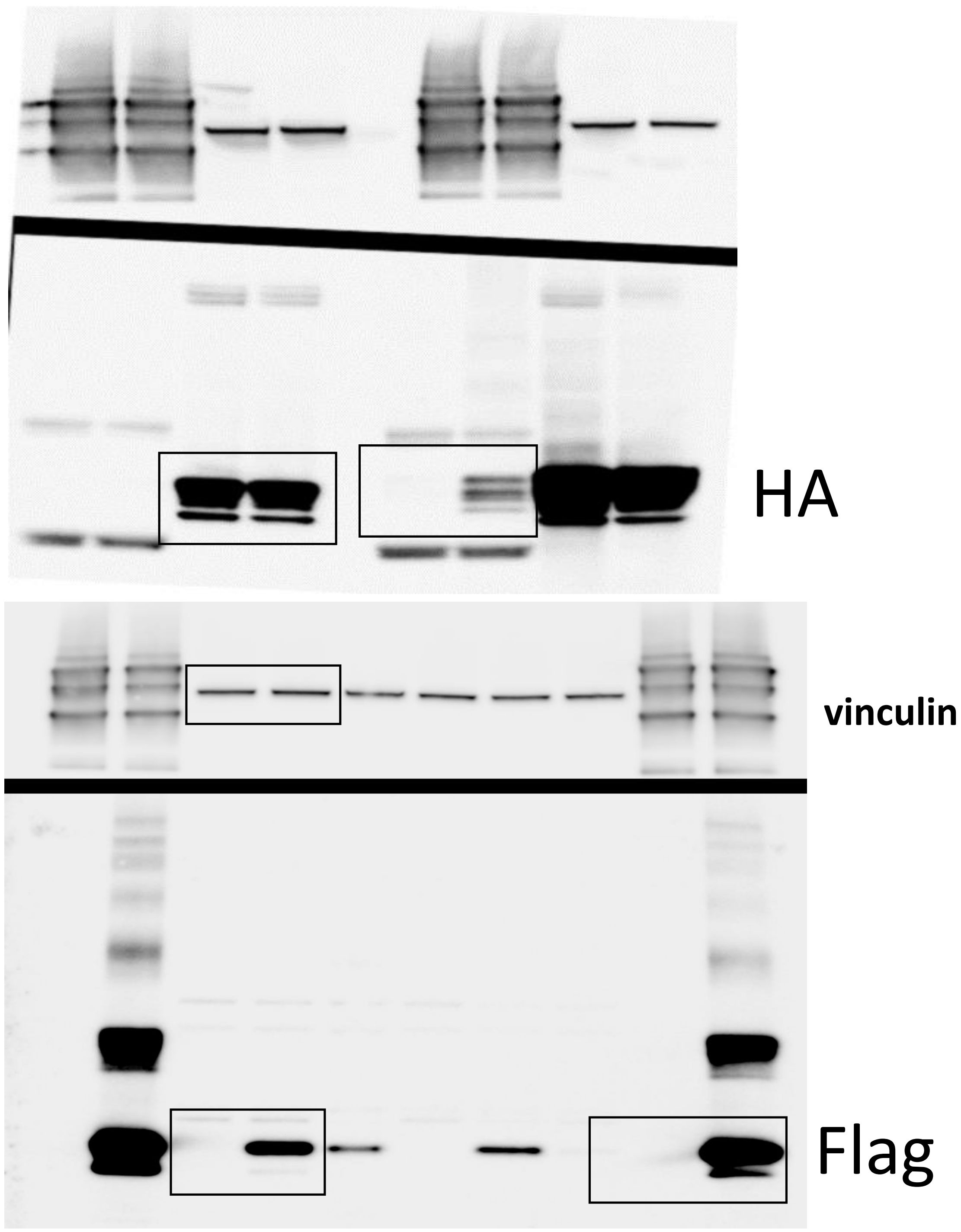

Supplement: Supplementary file 5 — Source Data for Figure 1 [file EMMM-8-288-s004.pdf]
