# Supplementary material for: OTUB1 triggers lung cancer development by inhibiting RAS monoubiquitination
Source: EMBO Mol Med. 2016 Feb 8;8(3):288–303. doi: 10.15252/emmm.201505972 (PMC4772950; doi:10.15252/emmm.201505972)

Full unedited gels for Figure 3A

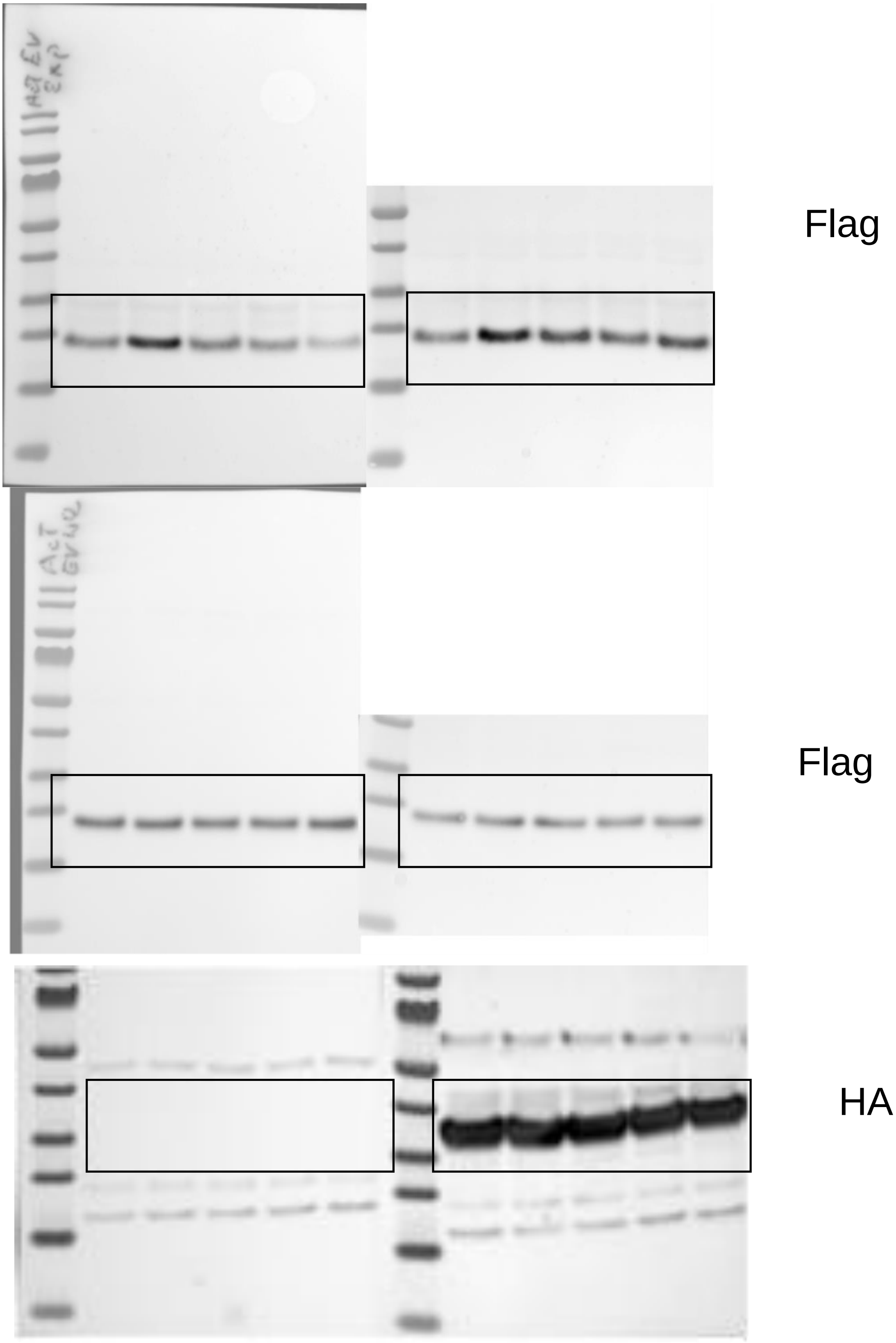

Full unedited gels for Figure 3B

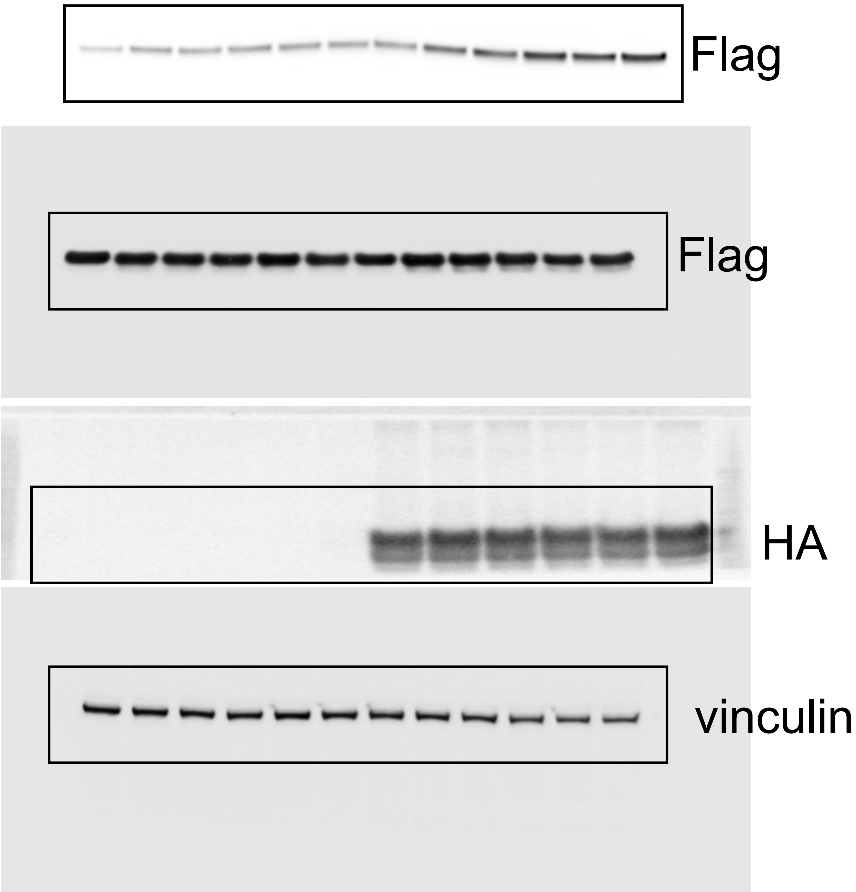

Full unedited gels for Figure 3E

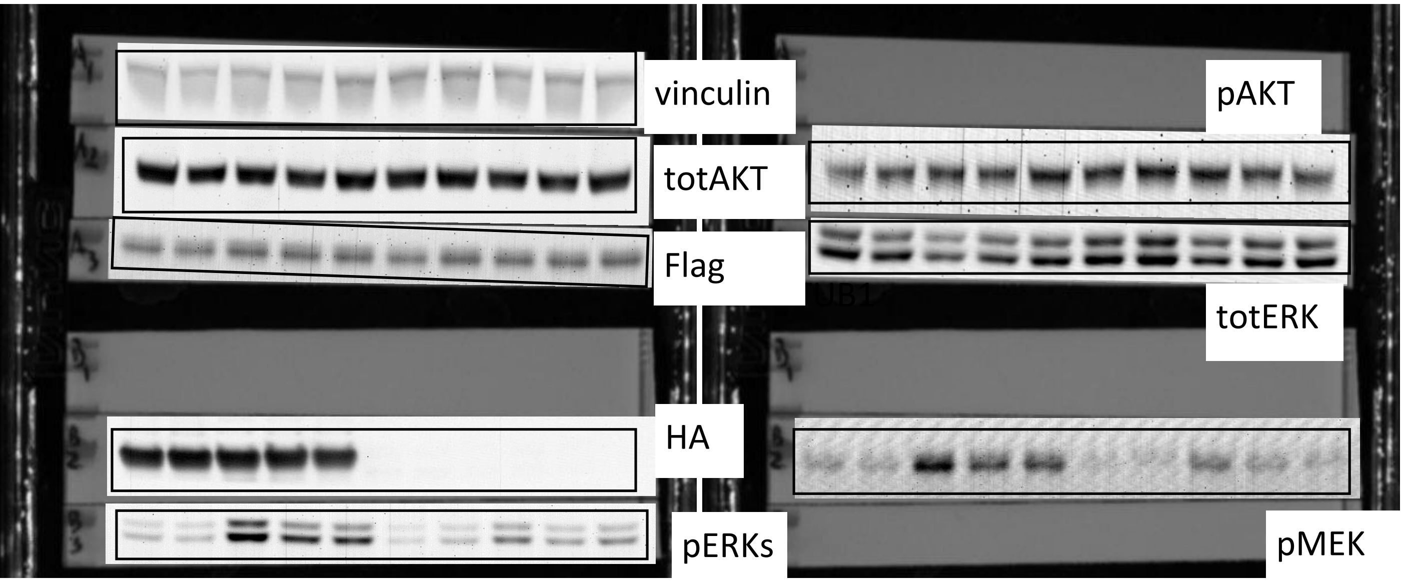

Full unedited gels for Figure 3F

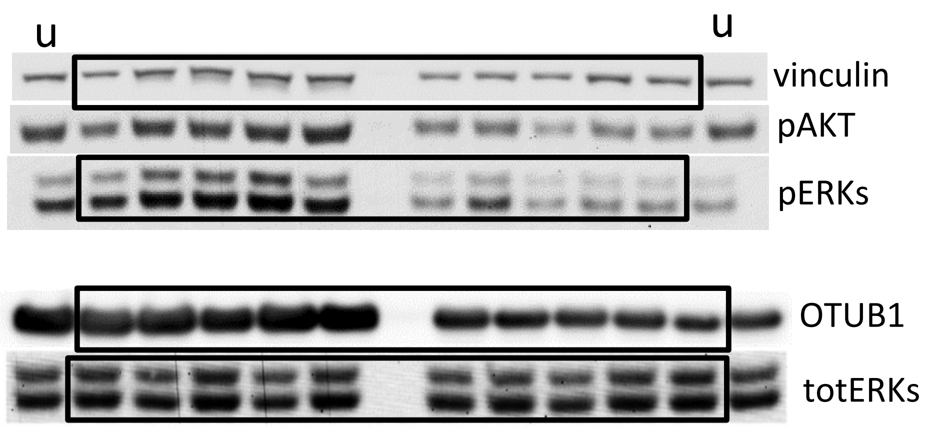

Full unedited gels for Figure 3G

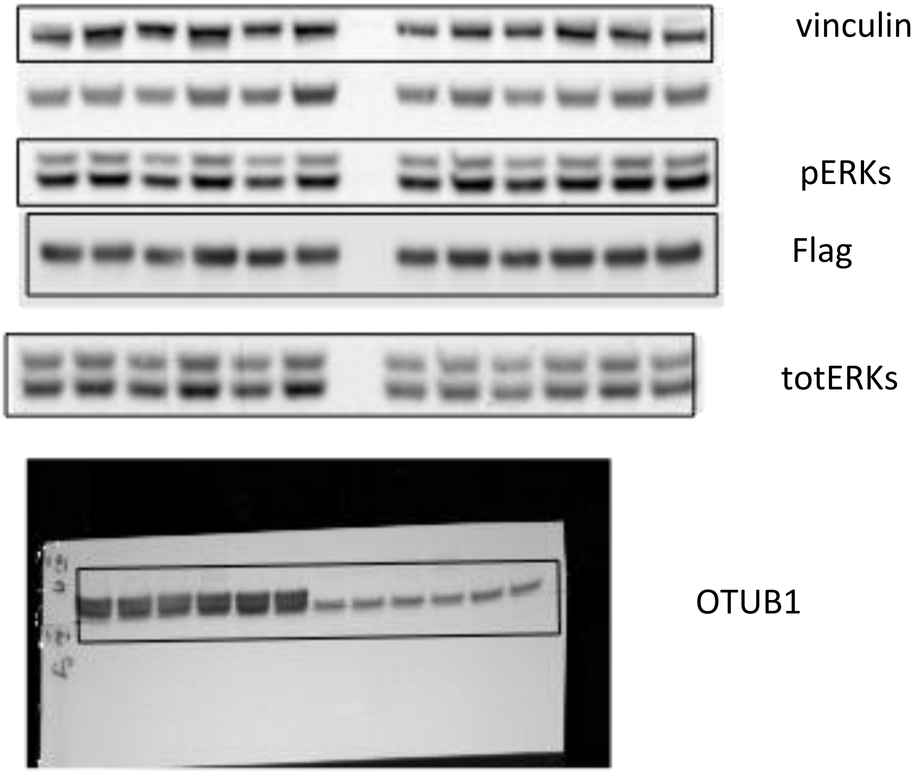

Full unedited gels for Figure 3H

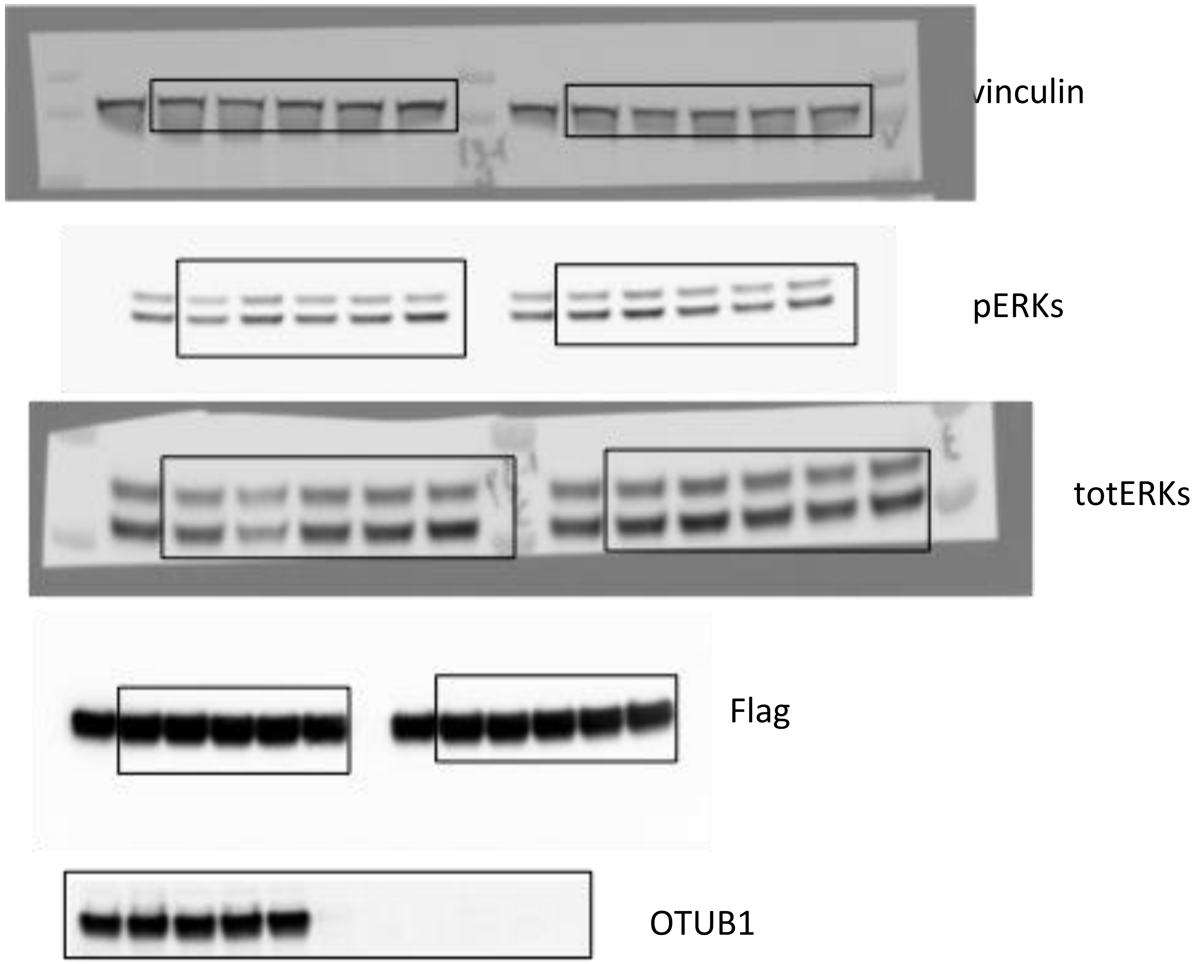

Full unedited gels forFigure 3I

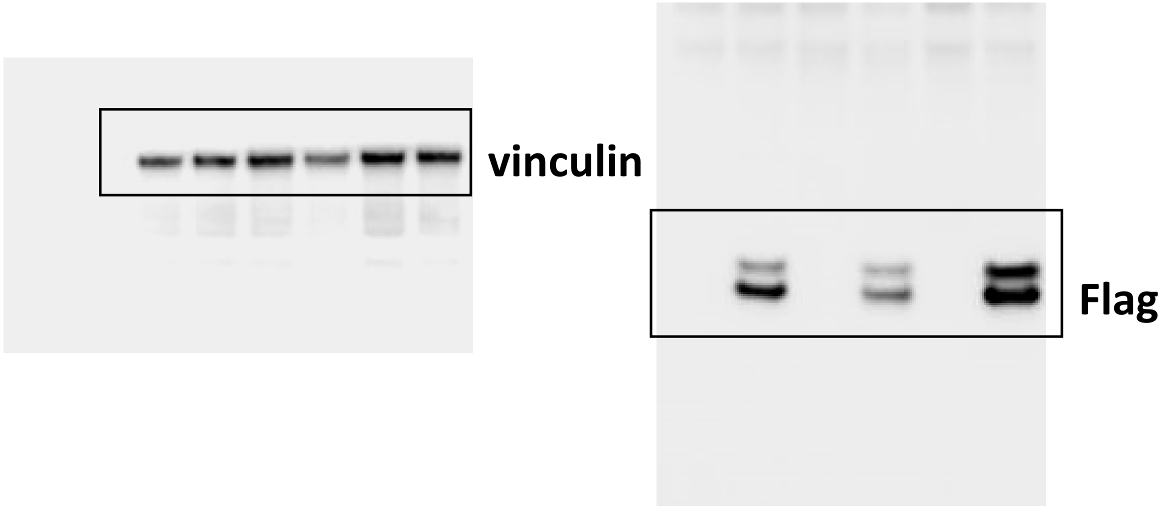

Supplement: Supplementary file 7 — Source Data for Figure 3 [file EMMM-8-288-s006.pdf]
